# Correlation Between the New Bite Force Meter and GC Dental Prescale II

**DOI:** 10.1111/joor.70173

**Published:** 2026-03-02

**Authors:** Mineka Yoshikawa, Yutaro Takahashi, Shion Maruyama, Azusa Haruta, Sachiko Yamada, Mariko Maruyama, Maho Takeuchi, Miyuki Yokoi, Mitsuyoshi Yoshida, Kazuhiro Tsuga

**Affiliations:** ^1^ Department of Advanced Prosthodontics Graduate School of Biomedical and Health Sciences Hiroshima University Hiroshima Japan; ^2^ Speech Clinic, Division of Specific Dentistry Hiroshima University Hospital Hiroshima Japan; ^3^ Department of Dentistry and Oral‐Maxillofacial Surgery Fujita Health University Toyoake Japan

**Keywords:** bite force, bite force meter, dental occlusion, dental Prescale II, healthy older adults, oral function

## Abstract

**Background:**

Measurement of bite force is important for understanding oral function.

**Objectives:**

We aimed to clarify the relationship between the values of a newly developed one‐sided measurement‐type bite force meter (OBM‐01 Prototype, JMS, Hiroshima) and those of a full‐dentition bite force meter (Dental Prescale II, GC, Tokyo). Furthermore, we examined the intra‐ and inter‐rater reliability (intraclass correlation coefficients; ICCs) using the OBM‐01 Prototype.

**Methods:**

Three studies were conducted. The first examined the ICC of the OBM‐01 Prototype in 22 healthy adults; the second evaluated the relationship between the habitual chewing side bite force measured by the OBM‐01 Prototype in 343 older adults, with or without dentures; and the third assessed the relationship between measurements obtained from the OBM‐01 Prototype and Dental Prescale II in 201 older adults.

**Results:**

The ICCs showed good reliability. A moderate correlation was observed between the OBM‐01 Prototype and Dental Prescale II, regardless of sex or denture presence on the habitual chewing side (Spearman's Rho; men 0.65, women 0.51 in non‐denture group, men 0.51, women 0.55 in denture group). In both men and women, compared with the non‐habitual chewing side, the habitual chewing side had significantly greater bite force. Additionally, the bite forces on the habitual and non‐habitual chewing sides were strongly and positively correlated with the total bite force measured using the Dental Prescale II (Spearman's Rho; men: habitual 0.676, non‐habitual 0.746, women: habitual 0.705, non‐habitual 0.640).

**Conclusions:**

A correlation was observed between the measurements obtained from the OBM‐01 Prototype and Dental Prescale II.

## Graphical abstract

This study evaluated a new one‐sided bite force meter (OBM‐01). In healthy and older adults, the device showed good reliability and moderate correlation with Dental Prescale II measurements. Habitual chewing side bite force was significantly higher, suggesting OBM‐01 may be useful for assessing oral function in clinical settings.

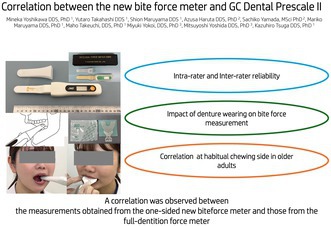

## 1 Introduction

The concept of oral frailty, which originated in Japan [1], is gaining global recognition. To address this issue, detailed oral function tests have been developed as standardised methods for assessing oral frailty, with the condition being termed as oral hypofunction [2]. These detailed oral tests are used in clinics and for frailty prevention in the community. Bite force measurement is one of these oral tests, and although a full‐dentition‐type bite force meter using a pressure‐sensitive sheet is generally used in clinical practice, the measurement process requires preparation, making it less practical for use in community screening settings.

**FIGURE 1:**
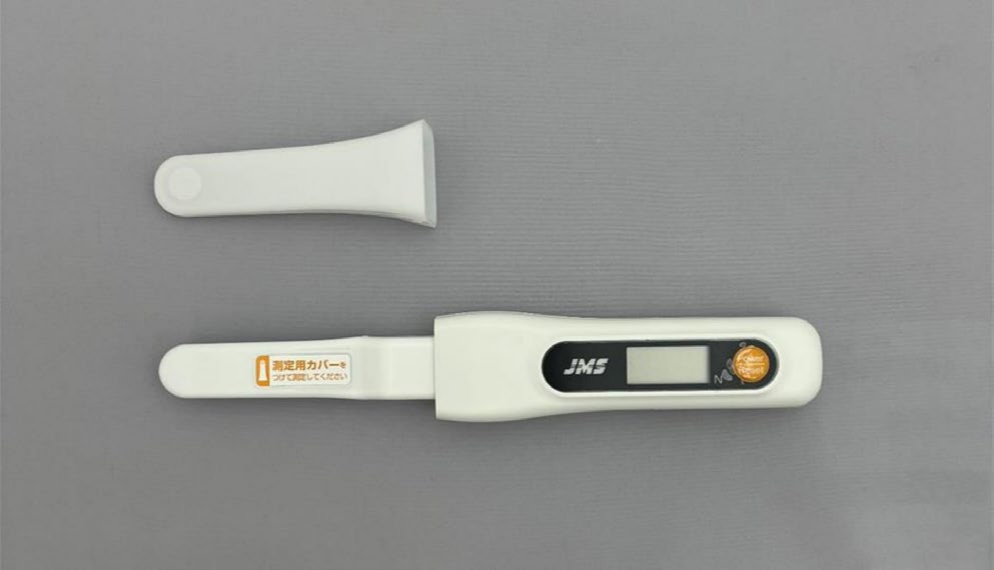
OBM‐01 prototype.

In contrast, a one‐sided bite force meter that measures the bite force at only one point between the maxilla and mandible is a simpler device. Recently, the JMS bite‐force meter (OBM‐01 Prototype, JMS, Hiroshima, Japan; Figure 1) was approved as a medical device (medical device notification number 34B1X00001000124), and it is now commercially available. This device measures the pressure of a liquid enclosed within a bite pressure gauge using a pressure sensor. When the molars bite down on the sensor, which is approximately 10 mm thick including its cover, the bite force is instantly displayed on a liquid crystal display on the main unit. This device is small, lightweight, and easy to use; therefore, it can be used not only by dental care workers but also by other medical professionals. However, since the measurement is taken in a raised bite position, limitations arise, such as the inability to measure the bite force in the intercuspal position and uncertainty whether the recorded values accurately reflect the actual bite force of the entire dentition.

**FIGURE 2:**
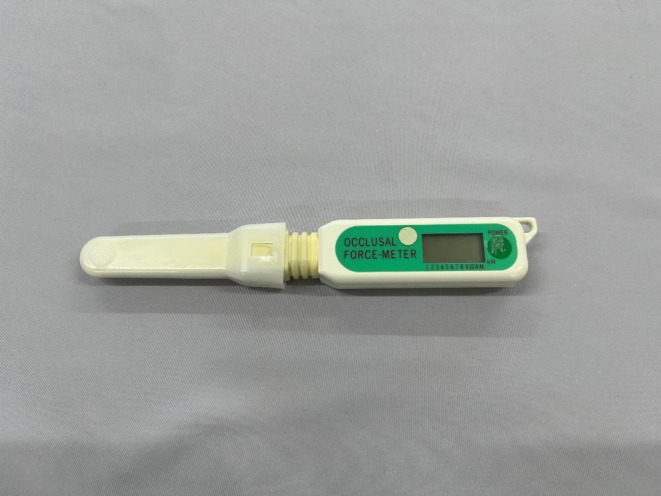
GM‐10.

The GM‐10 (Nagano Keiki, Tokyo; Figure 2), a device used for measuring the bite forces of individual teeth, is a unique and useful tool for oral function assessment. It easily measures bite force on individual teeth, instantly presenting the results. Its simplicity and immediacy, in particular, can significantly contribute to improving patient motivation and visualising treatment effects. However, production of the GM‐10 has been discontinued in recent years. Therefore, a simple device similar to the GM‐10 that can measure the bite force of individual teeth and serve as a surrogate to represent an individual's oral function is needed in research and clinical settings. The OBM‐01 Prototype is functionally a successor of the GM‐10. It differs from the GM‐10 in the material of its disposable cover and in the accuracy of its measurements. In this study, we aimed to examine the utility of the OBM‐01 Prototype by determining whether bite force measurements obtained with the OBM‐01 Prototype correlate with those from a full‐dentition bite force meter.

## 2 Methods

### 2.1 Bite Force Meters

The Dental Prescale (DP) II (GC Corporation, Tokyo) was used as the full‐dentition‐type bite force meter. The DP II uses a sheet‐type pressure‐sensitive film in which microcapsules containing a colouring agent break down and change colour depending on the magnitude of external force, and the colour changes depending on the strength of the applied force. The colour is read using a dedicated analytical device, and the left–right balance of the occlusal contact area, occlusal pressure, and bite force can be evaluated [3, 4].

**FIGURE 3:**
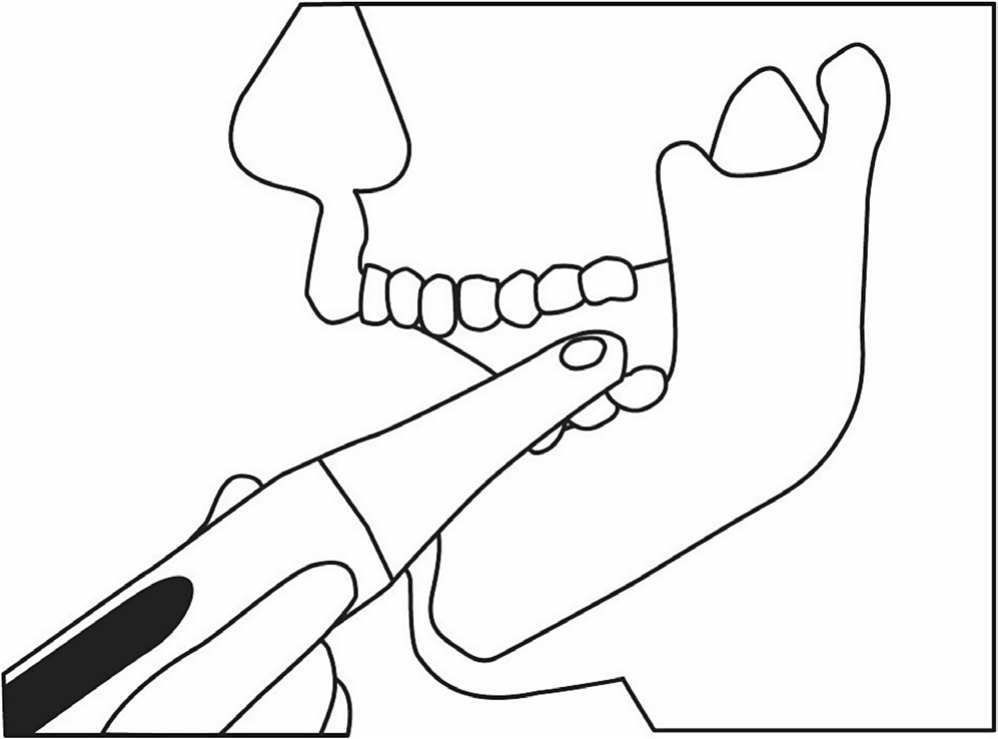
Image of the measurement site.

The one‐sided measurement‐type bite force meter used was the OBM‐01 Prototype, which consists of a main body for performing the measurements and a disposable measurement cover (Figure 1). The main body is equipped with a liquid crystal display and a power/reset button. When a participant bites the occlusal area, pressure is applied to the pressure sensor through the liquid inside and is converted into an electrical signal. This signal is amplified, A/D converted, and sent into a microcomputer, which converts the value into a bite force value and displays it on a display screen. Before the measurements, the examiner verbally instructed the participants about the risk of damaging their teeth and to stop biting immediately if they experienced any dental pain or discomfort. During the measurement, the measurement cover is firmly pressed onto the probe of the main body and attached. After pressing and holding the power/reset button for 2 s to turn on the power, a buzzer sounds, the display screen lights up, the entire display appears, and the device automatically enters standby mode. The device is then inserted into the mouth of the participant and positioned over the first molar (Figure 3). The measurement tip is placed on the central fossa of the mandibular first molar, approximately parallel to the occlusal surface of the first molar. Thereafter, the participant is instructed to gradually bite down on the sensor as hard as possible without feeling pain. The participant is instructed to stop biting on the sensor after the buzzer beeps three times (“pipipipi”). The device is then removed from the mouth, and the measurement is complete. The maximum bite force is then displayed on the screen. When the measurement is complete, the measurement value changes from flashing to being constantly illuminated. There is also a built‐in safety feature that emits a long beep if the bite force exceeds 700 N. The bite force was measured three times using the one‐sided bite force meter on both the habitual and non‐habitual chewing sides. To determine the habitual chewing side, the dentists placed a sterilised cotton ball with a diameter of approximately 7 mm on the dorsum of the tongue in the mouth and instructed the participants to bite naturally [5, 6]. After two measurements, if the chewing side was the same, it was determined to be the habitual chewing side. If two measurements were performed separately, a third measurement was performed, and the side with multiple measurements was determined to be the habitual chewing side. In this study, we carried out each measurement at room temperatures between 23°C and 26°C.

### 2.2 Intra‐Rater and Inter‐Rater Reliability

The reproducibility of the bite force measurement was examined using 22 healthy volunteers (25–48 y, 12 men, 10 women), in whom the measurement was performed three times using the identical procedure on separate occasions with 1 week between measurements; all participants provided written informed consent. Participants were excluded if they had a missing first molar (those who had not been treated with dentures) or occlusion problems such as temporomandibular joint disorders, or if they were undergoing orthodontic treatment. The sample size was determined based on previous studies [7]. All participants were asked to stop biting on the sensor immediately if they felt pain or discomfort. No participant reported pain during or after the measurement. Three dentists with at least 3 y of experience in prosthodontics served as raters. They evaluated the reproducibility of the device on the same 22 volunteers, in whom the bite forces were measured using the exact same procedure each time. Prior to data collection, the raters underwent a standardised training session to ensure consistent application of the measurement protocol. Each participant was assessed three times by Raters 1–3. For assessing intra‐rater reliability, the second assessment by Rater 1 was conducted approximately 1 week after the first assessment. For assessing inter‐rater reliability, Raters 1–3 independently assessed each participant on different days, with intervals of more than 1 week between assessments. Raters were blinded to each other's scores and their own previous scores to prevent recall bias.

### 2.3 Effects of Denture Wearing on Bite Force Measurement

We clarified whether the one‐sided bite force measured using the OBM‐01 Prototype correlated with the total bite force measured using the DP II, regardless of whether dentures were present in the oral cavity. The measurement of bite force with the OBM‐01 Prototype on the habitual chewing side was repeated three times. Dentures were inserted before measurements if the participant used them. This study involved older adults aged ≥ 60 y who participated in a survey and provided written informed consent to participate. Three hundred and forty‐three adults (57 men and 286 women, 60–91 y) participated in this study and were categorised into non‐denture (30 men, 179 women) and denture groups (27 men, 107 women).

The non‐denture group included participants who did not wear dentures at all, and the denture group included those who wore one or more removable dentures in the oral cavity, regardless of whether they had a missing first molar on the habitual chewing side or used an oral prosthesis. If these experiments showed correlation, this would indicate that the bite force of individual teeth measured using the OBM‐01 Prototype could potentially serve as a representative indicator of a person's oral function, even if the patient uses an oral prosthetic device.

### 2.4 Correlation of Bite Forces in the DP II vs.OBM‐01 Prototype at the Habitual Chewing Side in Older Adults Aged > 75 y

We aimed to determine whether the bite force measured using the OBM‐01 Prototype correlated with the total bite force measured by the DP II. This study involved older adults aged ≥ 75 y who participated in a survey. The study participants provided written informed consent to participate. We only included participants who had a first molar on the habitual chewing side as a natural tooth or denture (including implants and pontics). When it was not possible to measure the bite force using the upper and lower first molars, the force was measured at the position of the upper second premolar and lower first molar where maximum voluntary clenching could be performed. The exclusion criteria included participants with a missing first molar (not treated with dentures) and those with occlusion problems such as temporomandibular joint disorders. In total, 201 older adults (38 men and 163 women, 76–81 y) participated in this study.

### 2.5 Correlation of Masticatory Function and Bite Forces in the DP II and OBM‐01 Prototype in Older Adults

We clarified whether the effectiveness of bite force using OBM‐01 Prototype and DP II can assess the masticatory function. Masticatory function was assessed using glucose concentration obtained from chewed gummy jelly (Glucolumn, GC Corporation, Japan). Participants hold 10 mL water in their mouth for few seconds and spit into a cup with a plastic mesh filter. After chewing gummy jelly for 20 s. The amount of glucose was measured using a masticatory ability testing system (Gluco Sensor GS‐2, GC Corporation, Tokyo, Japan) [2]. In total, 132 older adults (26 men and 106 women, 72.8–82.5 y) participated in this study.

### 2.6 Statistical Analysis

Intra‐ and inter‐rater reliability were assessed using intraclass correlation coefficients (ICCs). Based on the study design, a one‐way random effects model with agreement (ICC [1] and ICC [1, 3]) for both intra‐rater reliability and a two‐way random effects model with absolute agreement (ICC [1, 2] and ICC [2, 3]) were chosen for inter‐rater reliability analyses, as raters were considered random samples and the goal was to assess absolute agreement between scores. Specifically, a two‐way random effects model with absolute agreement (ICC [1, 2]) was employed for both analyses. The interpretation of ICC values was based on the guidelines proposed by Koo and Li [8]: < 0.50 poor, 0.50–0.75 moderate, 0.75–0.90 good, and > 0.90 excellent.

Comparisons of bite forces measured using the OBM‐01 Prototype between men and women and between the habitual and non‐habitual chewing sides for each sex were performed using the Mann–Whitney U test. The correlation between the bite forces measured using the OBM‐01 Prototype on the habitual and non‐habitual chewing sides and the total bite force measured using the DP II for each sex was examined using Spearman's rank correlation coefficient. Furthermore, the correlation between the bite forces on the left and right sides measured using the DP II was examined. Also, the correlation between masticatory function and the bite forces using the OBM‐01 Prototype on the habitual chewing sides or the total bite force measured using the DP II for each sex was examined using Spearman's rank correlation coefficient. Statistical analyses were performed using SPSS software (ver. 23; IBM Corp., Armonk, NY), with a significance level of 5%.

This study was approved by the ethics committee of Kyoto University of Advanced Science (#24 M02) and the ethics committee of Hiroshima University (E2023‐0302). It was conducted in accordance with the guidelines of the national government based on the Helsinki Declaration of 1964. Written informed consent was obtained from all participants.

## 3 Results

### 3.1 Intra‐and Inter‐Rater Reliability

The intra‐rater reliability for Rater 1, as assessed using ICC(1,1) was 0.850 (95% confidence interval [CI]: 0.726–0.929), indicating good reliability, while that by ICC(1,3) was 0.945 (95% CI: 0.888–0.975), indicating excellent reliability. Additionally, the same good or excellent reliability was found for Raters 2 and 3 (Table 1). The inter‐rater reliability at the first measurement, as assessed using ICC(2,1) was 0.767 (95% CI: 0.592–0.886), indicating good reliability. In addition, that using ICC(2,3) was 0.908 (0.813–0.959), showing excellent reliability (Table 1).

**TABLE 1:** Inter‐ and intra‐rater reliability.

Inter‐rater reliability	ICC (1, 1) 95% CI	ICC (1,3) 95% CI
Rater 1	0.850 (0.726–0.929)	0.945 (0.888–0.975)
Rater 2	0.898 (0.808–0.953)	0.964 (0.927–0.984)
Rater 3	0.856 (0.735–0.932)	0.947 (0.893–0.976)

Intra‐rater reliability	ICC [2,1] 95% CI	ICC [2,3] 95% CI
First time	0.767 (0.592–0.886)	0.908 (0.813–0.959)
Second time	0.826 (0.687–0.917)	0.935 (0.868–0.971)
Third time	0.808 (0.657–0.907)	0.926 (0.852–0.967)

### 3.2 Impact of Denture Wearing on Bite Force Measurement

Three hundred and forty‐three participants were categorised into two groups: 209 adults (30 men and 179 women) in the non‐denture group and 134 adults (27 men and 107 women) in the denture group. The occlusion status of the first molar on the habitual chewing side in the denture wearer group is shown in Table 2. Two participants had missing maxillary first molars; however, due to the alignment of the teeth, it was possible to measure the occlusion using the adjacent second premolar instead of the mandibular first molar (Table 3). The denture group showed lower values than the non‐denture group. A moderate correlation was observed between the OBM‐01 Prototype and the DP II, regardless of sex or the presence of dentures (Table 3). No statistical differences were noted between men and women in the non‐denture group using the OBM‐01 Prototype or between men and women in the non‐denture group and men in the denture group using the DP II.

**TABLE 2:** Relationship between maxilla and mandible teeth on the habitual chewing side when measuring bite force with OBM‐01 Prototype.

	33 Adults	42 Adults	31 Adults	26 Adults	2 Adults
Maxilla	Remaining first molar	Dentures artificial teeth	Remaining first molar	Dentures artificial teeth	Second Premolar
Mandible	Remaining first molar	Dentures artificial teeth	Dentures artificial teeth	Remaining first molar	Remaining first molar

**TABLE 3:** Bite force measured using the Dental Prescale II (total force) and OBM‐01 Prototype at the habitual chewing side for non‐denture and denture groups.

	No.	Age	No. of remaining teeth	DP II total force (N)	OBM‐01 Prototype (*N*)	Spear man's Rho (*ρ*) between DPII and OBM‐01 Prototype
Non‐denture group	209					
Men	30	78.5 (73–81)	28 (25–28)	1321.1 (929.9–1753.8)	562.5 (392.5–690.5)	0.65*
Women	179	76.0 (73–80)	26 (25–28)	818.6 (603.5–1094.2)	474.0 (246.0–569.0)	0.51*
Denture group	134					
Men	27	81 (77–84)	21 (7–25)	780.0 (455.2–1027.1)	295.0 (192.0–438.0)	0.51*
Women	107	80 (76–83)	17 (12–21)	443.7 (292.2–749.9)	195.0 (122.0–299.0)	0.55*

*Note:* Presented as the median (Interquartile range). OBM‐01 Prototype, JMS Bite Force Meter Prototype.

Abbreviations: DP, Dental Prescale; No, Number.

* *p* < 0.05.

### 3.3 Bite Force by the DPII vs. Bite Force by the OBM‐01 Prototype

A total of 201 people (38 men and 163 women) aged ≥ 75 y underwent testing using the two types of bite force meters. The measurements are presented in Table 4. The results from using the OBM‐01 Prototype on the habitual and non‐habitual chewing sides were not significantly different between men and women. Additionally, the habitual chewing side showed higher values than the non‐habitual chewing side in both men and women.

**TABLE 4:** Bite force measured using the Dental Prescale II (total force) and OBM‐01 Prototype in 201 participants.

		No.		*P* (two‐tailed)
	Men	38	79 (76–81) y	0.357
	Women	163	80 (76–81) y	
Remaining teeth(*N*)^a^	Men	38	25 (22–28)	0.156
	Women	163	24 (18–27)	
DPII^a^				
Force Right (*N*)^a^	Men	38	415.2 (269.4–750.0)	0.002*
	Women	163	320.5 (185.1–495.1)	
Force Left (*N*)^a^	Men	38	549.6 (312.3–731.7)	< 0.001*
	Women	163	346.4 (196.5–498.9)	
Force Total (*N*)^a^	Men	38	987.5 (654.6–1379.5)	< 0.001*
	Women	163	714.1 (394.6–957.4)	
OBM‐01 PROTOTYPE (*N*)				
Habitual chewing side^a^	Men	38	422.0 (280.8–599.8)	0.039*
	Women	163	348.0 (197.0–528.0)	
Non‐habitual chewing side^a^	Men	36	320.0 (207.8–567.3)	0.194
	Women	155	281.0 (167.0–490.0)	

*Note:* OBM.‐01 Prototype, JMS Bite Force Meter Prototype.

Abbreviations: DP, Dental Prescale; No, Number.

^a^ Presented as the median (Interquartile range).

* *p* < 0.05.

In both men and women, Spearman's ρ showed a strong significant positive correlation between the total bite force using the DP II and both the habitual and non‐habitual chewing sides using the OBM‐01 Prototype (men; habitual: *ρ* = 0.676, *p* < 0.001, non‐habitual: *ρ* = 0.746, *p* < 0.001, women; habitual: *ρ* = 0.705, *p* < 0.001, non‐habitual: *ρ* = 0.640, *p* < 0.001; Table 5). Furthermore, a strong positive correlation was observed between the right‐sided bite force using the DP II and the right‐sided bite force using the OBM‐01 Prototype (36 men: *ρ* = 0.680, *p* < 0.001, 158 women: *ρ* = 0.697, *p* < 0.001) as well as between the left‐sided bite force using the DP II and the left‐sided bite force using the OBM‐01 Prototype (38 men: *ρ* = 0.802, *p* < 0.001, 160 women: *ρ* = 0.616, *p* < 0.001; Table 6).

**TABLE 5:** Correlation between Bite force the Dental Prescale II (total force) and OBM‐01 Prototype in habitual and non‐habitual chewing sides.

		DPII Total (*ρ*)	*P*
OBM‐01 Prototype			
Men	Habitual	0.676	< 0.001*
	Non‐habitual	0.746	< 0.001*
Women	Habitual	0.705	< 0.001*
	Non‐habitual	0.640	< 0.001*

*Note:* OBM‐01 Prototype, JMS Bite Force Meter Prototype, *ρ*: Spearman's Rho.

Abbreviation: DP, Dental Prescale.

* *p* < 0.05.

**TABLE 6:** Correlation between the Dental Prescale II (total force) and OBM‐01 Prototype in right and left sides.

		DP II Right		DP II Left	
		Men	Women	Men	Women
OBM‐01 Prototype					
Right	Men	*N* = 36, *ρ* = 0.680*			
	Women		*N* = 158, *ρ* = 0.697*		
Left	Men			*N* = 38, *ρ* = 0.802*	
	Women				*N* = 160, *ρ* = 0.616*

*Note:* OBM‐01 Prototype, JMS Bite Force Meter Prototype, *ρ*: Spearman's Rho.

Abbreviation: DP, Dental Prescale.

* *ρ* < 0.001.

### 3.4 Correlation of Masticatory Function and Bite Forces in the DP II andOBM‐01 Prototype in Older Adults

A total of 132 people (26 men and 106 women) aged ≥ 72 y underwent testing using the two types of bite force meters and masticatory ability testing system using gummy jelly. The measurements are presented in Table 7. In women, Spearman's ρ showed a significant positive correlation between masticatory function and the total bite force using the DP II, or the habitual chewing sides using the OBM‐01 Prototype (women; DP II: *ρ* = 0.477, *p* < 0.001, OBM‐01 Prototype: *ρ* = 0.445, *p* < 0.001; Table 8).

**TABLE 7:** Masticatory function and bite forces in the DP II andOBM‐01 Prototype in 132 older adults.

	No.	Age (y)	Masticatory function (mg/dL)	OBM‐01 Prototype habitual chewing side (*N*)	DP II Force Total (*N*)
Men	26	79 (72.8–82.5)	199.5 (160.5–221.5) y	328.0 (213.8–580.0)	7934.2 (457.5–1521.5)
Women	106	76 (73.8–80.0)	200.5 (165.0–233.5)	426.5 (246.8–566.8)	753.6 (515.9–1032.0)

*Note:* Presented as the median (Interquartile range). OBM‐01 Prototype, JMS Bite Force Meter Prototype.

Abbreviations: DP, Dental Prescale; No, Number.

**TABLE 8:** Correlation of masticatory function and bite forces in the DP II andOBM‐01 Prototype in older adults.

	Masticatory function Spear man's Rho (*ρ*)	
	Men	Women
OBM‐01 Prototype habitual chewing side (N)		
Men	*ρ* = 0.358	
Women		*ρ* = 0.445*
DP II Force Total (N)		
Men	*ρ* = 0.285	
Women		*ρ* = 0.477*

*Note:* OBM‐01 Prototype, JMS Bite Force Meter Prototype, *ρ*: Spearman's Rho.

Abbreviation: DP, Dental Prescale.

* *p* < 0.001.

## 4 Discussion

The bite force measurements obtained using the OBM‐01 Prototype were strongly and positively correlated with those obtained using the full‐dentition bite force meter DP II on both the habitual and non‐habitual sides. These findings suggest that the one‐sided measurement type can reliably reflect the overall bite force of an individual.

Based on prior clinical studies using the GM‐10, which can be considered the predecessor of the OBM‐01 Prototype, reliability varied widely from “poor” to “excellent” depending on the specific dental condition of the patient, the presence or absence of prostheses, and precise adjustment of the measurements in the mouth [9]. In this study, we fixed the occlusal surfaces of the first molars (including prosthetic devices such as dentures) on the habitual and non‐habitual chewing sides of the teeth as the measurement sites. Therefore, we believe that stable measurements were possible.

Factors that can significantly influence the ICC value include the sensitivity of the measurement position [10], the hardness of the measurement cover surface [11], and the lack of testing for environmental factors such as temperature and battery voltage [10]. In particular, relatively high reliability was observed among patients with some remaining teeth who were using removable dentures [9].

The performance of the OBM‐10 is highly dependent on the interaction of the operator and the subject, as well as biological variations. Therefore, the operator's skill, the subject's unique anatomy, and even environmental conditions may have a significant impact on the consistency of the measurements. Therefore, in addition to validating the device, the development of a comprehensive training program and adherence to strict clinical protocols will be essential to ensure consistent and reliable data across a variety of clinical settings. Due to its non‐invasive nature and simple ease of use, the OBM‐01 Prototype may become a valuable and widely available tool for objective bite force measurement in research and clinical practice. However, its maximum usefulness and validity of the measurements will depend on the user being fully aware of the factors that affect its reliability. These include the exact location of the device, the accuracy of the bite position with the cover, and the environmental settings during measurement, such as temperature. Continued research focused on optimising the measurement technique, addressing identified limitations, and further characterising the device's performance under a wider range of clinical conditions may improve the reliability of the OBM‐01 Prototype and expand its applicability in the developing fields of dentistry and medicine.

When evaluating the reliability of medical devices and evaluation methods, inter‐rater reliability is often given importance because verifying whether a device or method produces the same results regardless of who uses it (i.e., its “objectivity”) is essential for its practical use and widespread adoption in clinical settings. Therefore, while both figures are important, a high inter‐rater reliability strongly suggests that the evaluation method or device is more “robust” and suitable for widespread use. To improve inter‐rater reliability, it is important for developers and researchers to clarify the evaluation criteria and provide sufficient training to evaluators.

Furthermore, the effect of the measurement cover's surface material on maximum voluntary bite force measurements using the GM‐10 was investigated [10]. As a result, the original semi‐rigid bite surface of the device was replaced by a softer material (composed of leather and rubber), and consistently higher maximum voluntary bite force scores were recorded across both sexes and tooth groups. This means that the measurement cover's surface hardness directly influences the periodontal mechanoreceptor activation. Given the demonstrated position sensitivity [10] and the influence of bite surface hardness, it is essential to rigorously apply a standardised measurement protocol. This includes accurate and consistent intraoral setup (precise positioning of the cover measurement site on the mandibular first molar) and standardised patient instructions for biting. Therefore, a comprehensive training program for surgeons is required to minimise inter‐ and intra‐surgeon measurement errors and ensure overall reliability.

Bite force is generated on the occlusal surface of natural or artificial teeth when the mandible is raised by isotonic or isometric contraction, driven by jaw‐closing masticatory muscles. The jaw‐closing muscles that drive the bite force are the masseter, temporalis, and medial pterygoid muscles. When functional, these muscles are controlled by the third branch of the trigeminal nerve (mandibular nerve) and work together to generate bite forces of various magnitudes and directions. The amount of bite force that can be exerted varies depending on the area of action on the dentition. This phenomenon has not yet been fully elucidated because it involves the complex structure of the stomatognathic system. Moreover, most of the grinding and breaking down of food is carried out centrally in a limited area called the main functional area between the functional cusps of the first molar [12]. Some studies have examined the intermaxillary distance at which maximum bite force can be exerted in a wide range of participants, including adults, adolescents, and children. Reports vary regarding the intermaxillary location, such as the anterior teeth, canines, and molars. In adults, one study found no significant difference between intermaxillary distances of 2.5 mm and 6.0 mm at the first molar [13], while another reported that bite force could increase to an intermaxillary distance of 20 mm [14]. Arima et al. [15] performed their measurements using the GM‐10, the predecessor of the bite force meter used in the present study. They randomly raised the intermaxillary distance at the first molar area to 8, 12, 16, and 20 mm, and reported that 8 mm provided the maximum bite force. Therefore, we can assume that the optimal intermaxillary distance at which maximum bite force can be exerted is approximately 6–8 mm. The device used herein was 10 mm thick, including the disposable cover, so it is possible that the value shown was slightly smaller than the maximum value for the participant. In fact, the maximum bite force is the greatest on the first molar [16, 17, 18, 19], followed by the second molar, second premolar, first premolar, canine, and incisor; a study using a pressure‐sensitive sheet‐type bite force meter showed similar results. The bite force is greatest at the second molar, followed by the first molar, second premolar, first premolar, canine, and incisor in that order [20]. This implies that it is possible to roughly estimate an individual's bite force by measuring the bite force at the molars, which is supported by the results of this study.

It is believed that sex‐related bite force differences develop during the post‐puberty period in association with muscle development influenced by androgenic steroids in men [21, 22] and that a decline in bite force is associated with masticatory performance with aging [23]. Some investigations have established the influence of age and sex on maximum bite force [22, 24, 25]. Our findings were consistent with these results. Measurements were performed in triplicate, as multiple recordings are more reliable than a single recording of bite force [26]. The maximum value of the three measurements was used as the representative value for that participant.

Unilateral and bilateral bite forces are significantly correlated, and both measurement methods are suitable for evaluation of the functional state of the masticatory system [27]. The reason we focused on the correlation between the two types of bite force meters this time is that in Japan, only the sheet‐type devices can be used in medical settings as a medical device to measure a patient bite force [28, 29]. In addition to clinical settings, the GM‐10 can no longer be used in research and educational settings when a more handy and quick way to measure bite force is required. We felt that a comparison with the DP II was necessary to determine whether it was possible to visualise parts of the bite force. Given the moderate correlation, it cannot be said that the maximum bite force value of an individual tooth in the first molar region can be substituted for the total bite force of the entire jaw. This supports the conclusion of Srinivasan et al. [29] that the GM‐10 cannot be used “interchangeably” with the DP II and that it is inappropriate to directly calculate the bite force value of the entire jaw from the bite force value of an individual tooth. The results of Srinivasan et al. [30] differ slightly from our results, but this is thought to be due to factors such as the difference in the material of the disposable cover between the OBM‐01 Prototype and the GM‐10, the different ages of the participants, and the different numbers of participants. Based on this, we believe that although the “maximum bite force of an individual tooth in the first molar region” can be a predictor of the “total bite force of the entire jaw,” it is not possible to determine total jaw force by measuring individual teeth alone. Considering the usefulness of the OBM‐01 Prototype in assessing oral function, it may be better to also confirm its correlation with chewing ability. However, chewing ability is also related to tongue movement and saliva volume, among other factors, defined as the overall ability to chew and swallow food well. However, occlusal force can be said to be the “power” exerted when biting. Therefore, we conducted this study to measure this “power” and confirm its correlation with measurements using the known testing device DP II. One shortcoming of unilateral recordings is that, in denture wearers (mandibular first molars), loading only one side of the jaw may cause tilting of the prosthesis. Our participants using dentures did not report any discomfort or shifting of the prosthesis using the unilateral method.

One of the precautions to take when using the OBM‐01 Prototype is the risk of tooth damage. Before measurements began, participants were verbally informed of the risk of tooth damage and instructed to immediately stop biting if they experienced any tooth pain or discomfort. The device is equipped with a safety feature that sounds an alarm if the bite force exceeds 700 N. We do not know the reason for the limit of 700 N, but we speculate that it may be due to the following: Varga et al. [31] reported an average individual bite force in the first molar region of 18 year‐old men of 777.7 ± 78.7 N using the GM‐10. Considering the possibility that the OBM‐01 Prototype allows use with a variety of participants, we believe that it is appropriate to verbally explain the risks in advance. It is highly likely that this device will be used by people other than healthy adult men, such as older adults, women, and children. This device is a prototype, and the developers need to further consider measures to avoid the risk of tooth damage as much as possible. We believe that the results of this study support this.

In this study, OBM‐01 Prototype was employed for health checkups in areas where it was expected to be useful. Thus, it was possible to measure the bite force of adults aged > 75 y quickly (within a few minutes), allowing us to assess more than 400 participants over two 6‐h days. On the other hand, when using the DP II, it was necessary to scan the data obtained and check the overall bite force; thus, obtaining the test results took longer than when using the OBM. The results from using the DP II could not be communicated to the participants immediately, and they had to be notified by mail. Eto et al. investigated the relationship between occlusal pressure and fall risk among community‐dwelling older people, measuring occlusal pressure using the GM‐10 [32], which is no longer available for purchase. Yoshida et al. reported a relationship between postural stability and occlusion in older individuals with dementia [33]. Given the strong association between fall risk and postural stability, public health nurses actively working in the community have highlighted the usefulness of the GM‐10, emphasising its portability and the fact that measurements are non‐invasive, both psychologically and physically. From the perspective of care prevention and early detection of oral frailty in addition to general frailty, the GM‐10 has been recognised as useful, as it allows community‐dwelling individuals to assess aspects of their oral function not only using subjective evaluations such as “ease or of chewing” but also using objective measurements. We strongly support this approach and believe that the JMS‐01 Prototype can inherit these advantages.

In this study, we investigated the correlation between measurements obtained using a new device and DP II and masticatory function. Results indicated a moderate correlation only in 106 female participants. Furthermore, a correlation between DP II and masticatory function was also observed in these same female participants. Several reports exist regarding DP II and masticatory function [34, 35, 36, 37, 38, 39]. In a report on correlation using the masticatory function measurement method we used, using gummy jelly, Iyota et al. [34], who focused on older adults, confirmed this correlation using mixed‐gender data. Iyota et al. [34] surveyed participants aged 40 years or older who lived independently. Their report showed a correlation result (Speaman's Rho = 0.314) similar to ours. Although this masticatory function test using gummy jelly is widely used in various dental clinics and large‐scale surveys in Japan, there is still a paucity of reports specifically examining the correlation between total bite force measured by DP II and masticatory function. Sano et al. [35] reported that chewing ability might not be affected by aging as much as bite force, and Funahara et al. [36] reported that low tongue pressure is the only significant factor associated with decreased chewing ability. Chewing ability is thought to be influenced by a variety of factors, including saliva secretion ability, tongue movement, bite force, and number of remaining teeth, and we suspect that the “bite force measured at one point” reported here cannot be said to accurately assess masticatory function as a whole dentition.

This study had certain limitations. First, this study only collected data from older participants, and the number of men was relatively small. In addition, because measurements were primarily taken in older adults, it is necessary to investigate individual tooth bite forces in a larger‐scale survey of people of various ages, those with illnesses, and those requiring care. Second, the data were collected at two locations only. In the future, data collection at multiple locations will be necessary. Third, taking advantage of the OBM‐01 Prototype's ability to easily measure bite force, it is necessary to investigate bite forces at various sites (anterior teeth and premolars) and between alveolar ridges in edentulous individuals and to clarify the relationship to food crushing ability. Forth, One limitation of the 700 N warning level used in this study is that the rationale for its setting is not necessarily clear. The biomechanical resistance of teeth, dentition, prosthetic appliances, temporomandibular joints, and masticatory muscles varies greatly from person to person. Furthermore, it can vary significantly depending on a variety of factors, including age, tooth loss, denture fit, the condition of the denture‐supporting mucosa, decline in stomatognathic function, and cognitive function. Therefore, careful consideration is needed regarding the appropriateness of applying a uniform threshold as a warning standard. Furthermore, it should be noted that high occlusal forces that reach the warning level may carry potential risks, such as increased stress on periodontal tissues, tooth fracture, damage to restorative restorations and prosthetic appliances, and increased stress on the temporomandibular joint. In particular, excessive occlusal forces are more likely to cause clinical harm in elderly people and denture wearers due to tissue fragility and reduced occlusal support. From the above perspective, future research will require verifying the validity of the 700 N standard value, as well as more detailed safety considerations, such as setting a safety margin according to individual patient background, biomechanical simulation, and risk assessment during long‐term device use. It is also necessary to clarify the clinical validity of the warning level by continuously evaluating the effects of excessive bite force on actual clinical outcomes (pain, prosthetic breakage, masticatory efficiency, temporomandibular joint symptoms, etc.).

## 5 Conclusions

This study found a strong positive correlation between measurements obtained using one‐sided and full‐dentition bite force meters, and there were no adverse events such as tooth pain or chipping of teeth or dentures. Therefore, one‐sided bite force meters can be effectively used to promote oral health. We plan to conduct similar measurements in other age groups in future studies and to establish standard values. The OBM‐01 Prototype demonstrated good/excellent intra‐rater reliability and good inter‐rater reliability when used by trained raters in this study. These results support its use as a reliable tool for assessing oral function in clinical and research settings.

## Funding

KT received alumni association's support fund (Department of Removable Prosthodontics, Faculty of Dentistry, Hiroshima University). The other authors have declared that no other competing financial or nonfinancial interests existed at the time of publication.

## Conflicts of Interest

The authors declare no conflicts of interest.

## Data Availability

All data generated or analysed during this study are included in this published article.
